# On the Employment of Tartar Emetic to Relieve Rigidity of the Cervix and Os Uteri, in Cases of Parturition

**Published:** 1851-04

**Authors:** 


### RECORD OF MEDICAL SCIENCE.

### OBSTETRICS.

*On the employment of Tartar Emetic to relieve Rigidity of the Cervix and os uteri, in cases of Parturition.*—Dr. A. Hall, of Montreal, strongly recommends the employment of tartar emetic, to relieve rigidity of the cervix and os uteri, in parturition, and in *much larger doses* than has been advocated by writers on midwifery. Dr. Hall prescribes it in grain doses, every half hour. He has not found it necessary to exhibit the medicine in such doses more than thrice, relaxation most commonly following the exhibition of the first grain. Several cases are reported, strikingly illustrative of the value of the practice. Dr. Hall, however; admits that "cases may be met with, in which its exhibition would be decidedly improper. These exceptions to the rule will be found to occur in women of delicate habit and leucophlegmatic temperament. They are cases in which the action of the remedy, if exhibited, may proceed too far; which, unable to resist the prostrating effects of the medicine, might be followed by a collapse, to which the vital powers of the system might succumb. This is a contingency which should be sedulously kept in view; and prudence demands, therefore, some care in the selection of proper cases for its exhibition."—*Abridged from British American Medical and Physical Journal, December* 1850.

